# Supplementary figures and images for: Antibody Profiling and In Silico Functional Analysis of Differentially Reactive Antibody Signatures of Glioblastomas and Meningiomas
Source: Int J Mol Sci. 2023 Jan 11;24(2):1411. doi: 10.3390/ijms24021411 (PMC9866115; doi:10.3390/ijms24021411)

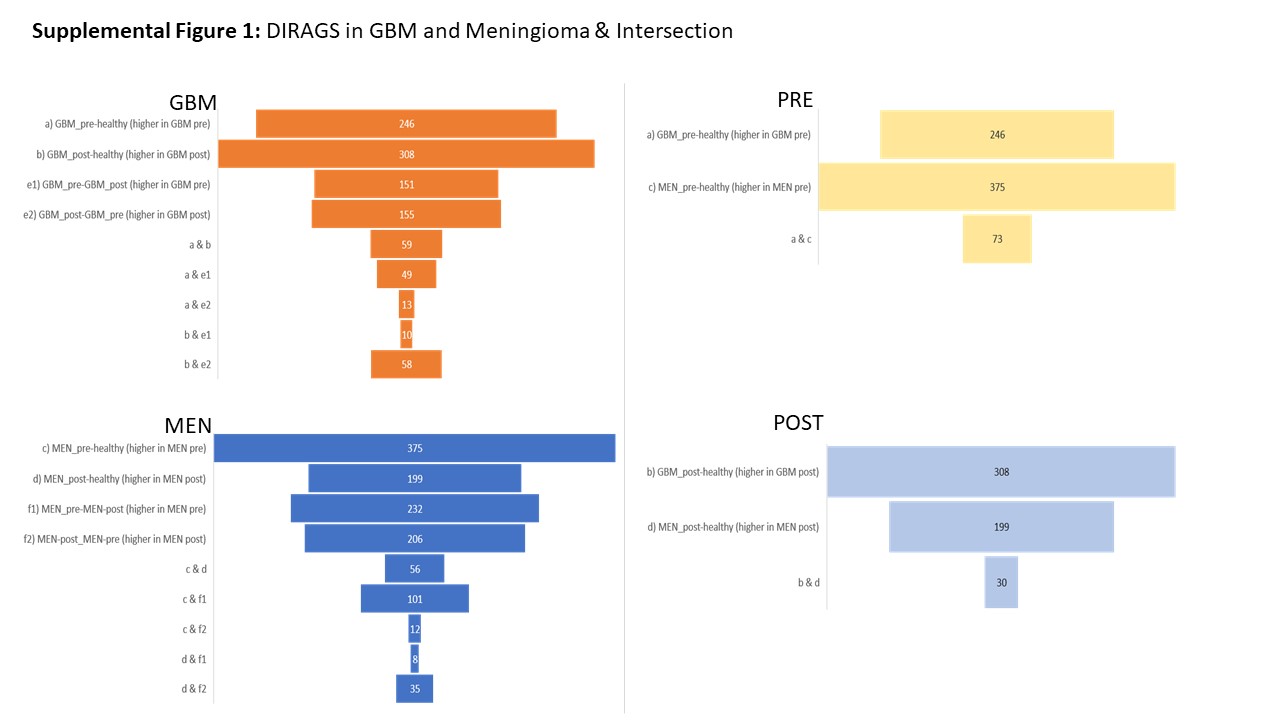

Supplement: Supplementary file 1 [file ijms-24-01411-s001.zip › Supplemental Figure 1.jpg]
